# Supplementary material for: Climate Change Influences Basidiome Emergence of Leaf-Cutting Ant Cultivars
Source: J Fungi (Basel). 2021 Oct 27;7(11):912. doi: 10.3390/jof7110912 (PMC8623619; doi:10.3390/jof7110912)
Supplement: Supplementary file 1 [file jof-07-00912-s001.zip › jof-1359651-supplementary.pdf]

Journal of Fungi

MDPI

*Supplementary material 1*

## Tables

**Table S1.** Field occurrences of basidiomes in leaf-cutting ant nests. Data reviewed in the literature were compiled with new occurrence reports since the review by Mueller (2002)[1].

| Ant Species | Fungal Species | Occurrence Data | Climate Data | Remarks from this Study | References |
|---|---|---|---|---|---|
| *Acromyrmex aspersus* | *Leucoagaricus gongylophorus* | Basidiomes were found on November 29, 1995, in an active nest in a native forest, in Santa Cruz do Sul-RS (Rio Pardinho), Brazil. | no data | Nest near a dead trunk, almost at the top of a hill. Basidiomes grew on an active nest in the field. No attempts of subculturing from basidiome or basidiospore germination were made. | [2] |
| *Acromyrmex coronatus* | *Leucoagaricus gongylophorus* | Basidiomes were observed on March 23, 2006, in Rio Claro-SP (UNESP Campus), Brazil. | no data | Bacci obtained basidiospores and tried to germinate them in culture. Germination was obtained but hyphae stopped growing and the colony did not develop. | Bacci M. (personal communication) |
| *Acromyrmex coronatus* | *Leucoagaricus gongylophorus* | Basidiomes were observed on January 5 and 27, 2018, in Rio Claro-SP, Brazil (Colony id: BLS170701-01). | Basidiome occurrence in hot and rainy seasons | Active nest. No attempts of subculturing from basidiome or basidiospore germination were made. | This study |
| *Acromyrmex coronatus* | *Leucoagaricus gongylophorus* | Basidiomes were observed on April 5 and November 27, 2018, January 28, 2019, February 10, 2020, in Rio Claro-SP, Brazil (Colony id: RB181203-01). One specimen is deposited in the herbarium of the Federal University of Santa Catarina # FLOR0068416). | Basidiome occurrence in hot and rainy seasons | Active nest. No attempts of subculturing from basidiome or basidiospore germination were made. | This study |
| *Acromyrmex coronatus* | *Leucoagaricus gongylophorus* | Basidiome was observed on December 29, 2019, in Mogi-Guaçu-SP, Brazil (Colony id: RB200104-01). | Basidiome occurrence in hot and rainy seasons | Active nest. No attempts of subculturing from basidiome or basidiospore germination were made. | This study |
| *Acromyrmex coronatus* | *Leucoagaricus gongylophorus* | Basidiomes were observed on January 2 and 10, 2020, February 5 and 7, November 12, and December 13 and 18, 2020, in Rio Claro-SP, Brazil (Colony id: RB190909-01). | Basidiome occurrence in hot and rainy seasons | Basidiome subculturing resulting in mycelium with staphylae; Basidiospore germination was successfully attempted. | This study |
| *Acromyrmex coronatus* | *Leucoagaricus gongylophorus* | Basidiome was observed on January 4, 2020, in Mogi-Guaçu-SP, Brazil (Colony id: RB200104-03). | Basidiome occurrence in hot and rainy seasons | Active nest. No attempts of subculturing from basidiome or basidiospore germination were made. | This study |
| *Acromyrmex coronatus* | *Leucoagaricus gongylophorus* | Basidiome was observed on February 21, 2020, in Rio Claro-SP, Brazil (Colony id: RB200507-03). | Basidiome occurrence in hot and rainy seasons | Active nest. Basidiospore germination was successfully attempted. | This study |
| *Acromyrmex crassispinus* | *Rozites gongylophora (= Leucoagaricus gongylophorus)* | As mentioned in Gonçalves (1961, page 117) "Luederwaldt 1926 observed basidiomes on nests of A. crassispinus (cited as A. nigra). | no data | | [1,3,4] |
| *Acromyrmex disciger* | *Leucocoprinus gongylophorus (= Leucoagaricus gongylophorus)* | Basidiomes were observed in November 1891, February | no data | Basidiomes were completely grown after 10-12 days. Spore | [1,5,6] |

| | | | | | |
|---|---|---|---|---|---|
| | | 19, and March 17 and 30, 1892, in Blumenau-SC, Brazil. | | germination (limited number) was shown after two days on water or nutrient solution (unspecified). Fast and vigorous formation of gongylidia from pileo subculture and less so from spores. | |
| *Acromyrmex hispidus fallax* | *Rozites gongylophora (= Leucoagaricus gongylophorus)* | Observation from November 11, 1944, in Curitiba-PR, Brazil (see page 143). | no data | Basidiome was similar to Rozites gongylophora growing outside of a shallow nest of A. hispidus fallax. The base of the basidiome was formed by a rigid fungus garden, but with a conserved sponge-like aspect. | [1,3] |
| *Acromyrmex hispidus fallax* | *Leucoagaricus gongylophorus* | Last week of February 1996 (dozen of basidiomes) and April (ten basidiomes), in Rio Claro-SP, Brazil. The colony was found under a peach tree. | no data | Healthy and free-living nest (probably a field nest). | [1,7] |
| *Atta cephalotes* | Unidentified | no data/not sure | no data/not sure | | [1,8] |
| *Atta cephalotes* | Unidentified | Lelydorp, 20km near Paramaribo, Suriname. | no data/not sure | | [1,9,10] |
| *Atta cephalotes* | *Rozites gongylophora (= Leucoagaricus gongylophorus)* | November 8 and 9, 1939 in Paramaribo, Suriname (see page 253-254 + 258-266). | no data/not sure | | [1,11] |
| *Atta cephalotes* | *Rozites gongylophora (= Leucoagaricus gongylophorus)* | January 13, 1940, in Paramaribo, Suriname (see page 260). | no data/not sure | | [1,11] |
| *Atta colombica* | *Leucocoprinus cf. gongylophorus* | no data/not sure | no data/not sure | | Collection PA-236, U. G. Mueller, unpublished data |
| Not informed | *Rozites gongylophora* | Basidiome was observed on May 29, 1957. | no data | From soil. Recorded by J Rich. Voucher # URM 7583 (URM Culture Collection, Pernambuco, Brazil)[1] | gbif.org/occurrence/2464956731, last accessed on 25 June 2021 |
| We assume *Acromyrmex sp*. based on the collector's descriptions of ant colony | *Leucoagaricus gongylophorus* | Basidiome was observed on December 22, 2010, in Brasília-DF, Brazil. Sh Jardim Botânico/Condomínio Quintas Bela Vista Conjunto e - Jardim Botânico, Brasília - DF, 70297-400, Brasil (Coordinates: 15.9S and 47.8W). | no data | Ant nest made of plant matter and debris, located between palm trunks. | gbif.org/occurrence/1986496332, last accessed on 25 June 2021 |
| We assume *Acromyrmex sp*. based on the collector's descriptions of ant colony | *Leucoagaricus gongylophorus* | Basidiome was observed on November 11, 2017, in Parque Nacional da Serra dos Órgãos - Parnaso, Teresópolis-RJ, Brazil. | no data | Active nest covered with straw. No attempts of subculturing from basidiome or basidiospore germination were made. | Heisecke C., Duque J. & Venegas M. (personal communication) |

[1]The occurrence remarks "from soil" suggests that this might be a first and rare case of a free-living specimen, however, an official identification could not be confirmed.

**Table S2.** Results from Bartlett test of homogeneity of variances analysis.

| Climate Data | Bartlett's K-Squared | df | *p*-Value |
|---|---|---|---|
| Precipitation | 26,263 | 6 | 1,99E-04 |
| Minimal temperature | 16,417 | 6 | 1,17E-02 |
| Mean temperature | 38,239 | 6 | 1,01E-06 |
| Maximal temperature | 27,839 | 6 | 1,01E-04 |

**Table S3.** Results from PLS analysis and corresponding VIP scores.

| Predictors | Coefficient with Standard Error | t-value | *p*-Value | VIP Scores |
|---|---|---|---|---|
| Prep0 | 0,711 ± 0,614 | 1,19 | 0,253 | 0,158 |
| Prep1 | 0,934 ± 0,479 | 1,97 | 0,066 | 1,423 |
| Prep2 | −1,015 ± 0,615 | −1,70 | 0,108 | 1,265 |
| Prep3 | −0,002 ± 0,435 | 0,07 | 0,945 | 0,003 |
| Prep4 | 1,345 ± 0,608 | 2,19 | 0,044 | 1,819 |
| Prep5 | 0,355 ± 0,447 | 0,76 | 0,460 | 0,183 |
| Prep6 | −0,467 ± 0,473 | −0,98 | 0,340 | 0,066 |
| Tmax0 | 0,975 ± 0,591 | 1,63 | 0,122 | 0,328 |
| Tmax1 | 0,516 ± 0,554 | 0,89 | 0,385 | 0,017 |
| Tmax2 | 0,526 ± 1,726 | 0,37 | 0,714 | 1,609 |
| Tmax3 | −1,443 ± 1,200 | −1,28 | 0,218 | 0,532 |
| Tmax4 | −0,130 ± 0,364 | −0,30 | 0,768 | 1,209 |
| Tmax5 | −0,786 ± 2,004 | 0,33 | 0,746 | 1,092 |
| Tmax6 | −0,919 ± 2,017 | −0,40 | 0,698 | 1,169 |
| Tmean0 | 0,0901 ± 0,922 | 0,08 | 0,934 | 0,110 |
| Tmean1 | 0,727 ± 1,060 | 0,72 | 0,480 | 0,909 |
| Tmean2 | −0,826 ± 3,211 | −0,34 | 0,741 | 1,899 |
| Tmean3 | 0,690 ± 2,611 | 0,35 | 0,734 | 0,315 |
| Tmean4 | −1,124 ± 1,917 | −0,66 | 0,519 | 0,668 |
| Tmean5 | 2,031 ± 4,321 | 0,54 | 0,593 | 1,162 |
| Tmean6 | −1,391 ± 3,051 | −0,53 | 0,603 | 0,813 |
| Tmin0 | −0,600 ± 0,673 | −0,95 | 0,355 | 0,131 |
| Tmin1 | −0,199 ± 1,960 | −0,13 | 0,901 | 0,689 |
| Tmin2 | 1,272 ± 1,525 | 0,9 | 0,384 | 1,631 |
| Tmin3 | −0,755 ± 1,414 | −0,60 | 0,559 | 0,281 |
| Tmin4 | 0,175 ± 2,652 | 0,09 | 0,933 | 0,878 |
| Tmin5 | −0,476 ± 2,046 | −0,27 | 0,790 | 0,524 |
| Tmin6 | −0,607 ± 0,813 | −0,77 | 0,450 | 1,650 |

**Table S4.** Results from Kruskal-Wallis analysis.

| Predictors | Kruskal-Wallis Chi-Squared | df | *p*-Value |
|---|---|---|---|
| Precipitation | 44.682 | 6 | 5.41E+03 |
| Minimal temperature | 72.517 | 6 | 1.24E-06 |
| Mean temperature | 61.428 | 6 | 2.31E-11 |
| Maximal temperature | 35.776 | 6 | 3.05E-06 |

**Figures**

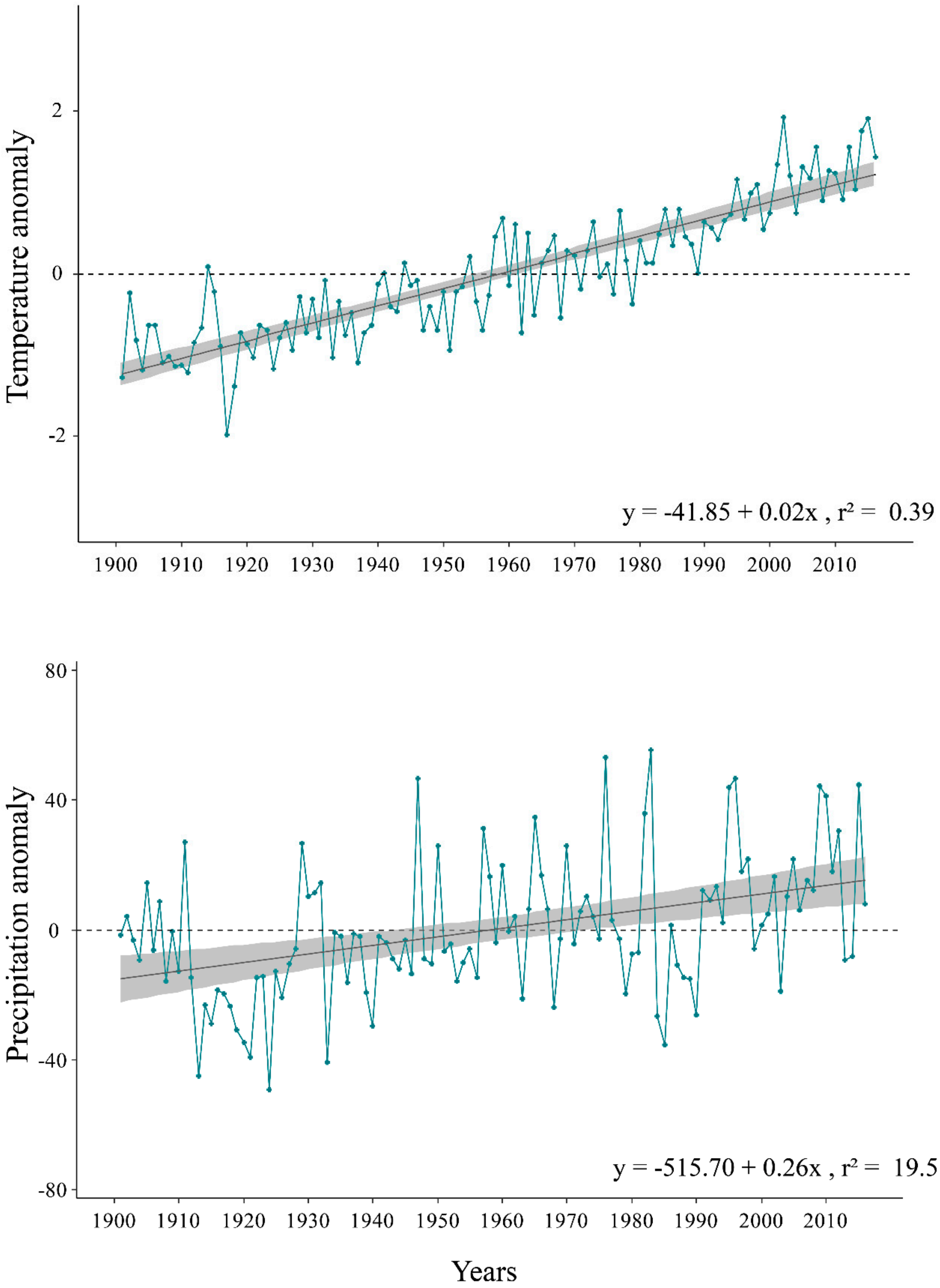


**Figure S1.** Historical climate data of Rio Claro obtained from World Bank Data (http://climateknowledgeportal.worldbank.org/; last accessed on 28 August 2020). Temperature and precipitation anomalies were calculated from an average from 1901 until 2016 (baseline; dashed horizontal line). A positive anomaly indicates higher temperature and precipitation than the baseline, while a negative indicates lower. The smooth grey area indicates how data fitted to a linear regression model (black line).

*Supplementary material 2*

**Video S1.** *Acromyrmex coronatus* ants transfer fungus gardens from belowground nests to nests in tree forks.

**Video S2.** *Acromyrmex coronatus* ants transferring fungus gardens with protective behavior against *Camponotus* sp.

## Reference

1. Mueller UG. 2002 Ant versus fungus versus mutualism: ant-cultivar conflict and the deconstruction of the attine ant-fungus symbiosis. *Am. Nat.* **160**, S67–S98. (doi:10.1086/342084)
2. Spielmann AA, Putzke J. 1998 *Leucoagaricus gongylophorus* (Agaricales, Basidiomycota) em ninho ativo de formigas Attini (*Acromyrmex aspersus*). *Cad. Pesq. Série Botânica* **10**, 27–36.
3. Gonçalves CR. 1961 O gênero *Acromyrmex* no Brasil (Hym. Formicidae). *Studia Entomol.* **4**, 113–180.
4. Luederwaldt H. 1926 Observações biológicas sobre formigas brasileiras especialmente do estado de São Paulo. *Rev. Museu Paul.***14**, 185–303.
5. Heim R. 1957 A propos du *Rozites gongylophora* A. Möller. *Rev. Mycol.* **22**, 293–299.
6. Möller A. 1893 Die Pilzgärten einiger südamerikanischer Ameisen, 1st ed. Jena, Verlag von Gustav Fischer, Germany, **6**, pp. 1–127
7. Pagnocca FC, Bacci Jr M, Fungaro MH, Bueno OC, Hebling MJ, Sant'Anna A, Capelari M. 2001 RAPD analysis of the sexual state and sterile mycelium of the fungus cultivated by the leaf-cutting ant *Acromyrmex hispidus fallax*. *Mycol. Res.* **105**, 173–176. (doi:10.1017/S0953756200003191)
8. Stahel G. 1938 Sobre o fungo cultivado pela formiga *Atta cephalotes* L. *An. Prim. Reun. Sul-Ameri. Bot.* **1**, 199–206.
9. Stahel G, Geijskes DC. 1939 Ueber den bau der nester von *Atta cephalotes* L. und *Atta sexdens* L. (Hym. Formicidae). *Rev. Entomol.* **10**, 27–78.
10. Stahel G, Geijskes DC. 1940 De parasolmieren en hunne bestrijding. *Bull. Depart. Landbouw.* **56**, 1–26.
11. Stahel G, Geijskes DC. 1941 Weitere Untersuchungen über den Nestbau und Gartenpilz von. *Atta cephalotes*. *Rev. Entomol.* **12**, 243–268.
